# The multiple molecular facets of fragile X-associated tremor/ataxia syndrome

**DOI:** 10.1186/1866-1955-6-23

**Published:** 2014-07-30

**Authors:** Chantal Sellier, Karen Usdin, Chiara Pastori, Veronica J Peschansky, Flora Tassone, Nicolas Charlet-Berguerand

**Affiliations:** 1 Department of Translational Medicine, IGBMC, INSERM U964 Illkirch, France; 2 Section on Gene Structure and Disease, NIDDK, National Institutes of Health, Bethesda MD 20892, USA; 3 Department of Psychiatry and Behavioral Sciences and Center for Therapeutic Innovation, Hussman Institute for Human Genomics, University of Miami, Miller School of Medicine, Miami FL 33136, USA; 4 Department of Biochemistry and Molecular Medicine, University of California, Davis, School of Medicine, Sacramento CA 95817, USA; 5 MIND Institute, University of California Davis Medical Center, Sacramento CA 95817, USA; 6 Institut de Génétique et de Biologie Moléculaire et Cellulaire, CNRS UMR7104, INSERM U964, University of Strasbourg, 1 rue Laurent Fries, Illkirch F-67404, France

## Abstract

Fragile X-associated tremor/ataxia syndrome (FXTAS) is an adult-onset inherited neurodegenerative disorder characterized by intentional tremor, gait ataxia, autonomic dysfunction, and cognitive decline. FXTAS is caused by the presence of a long CGG repeat tract in the 5′ UTR of the *FMR1* gene. In contrast to Fragile X syndrome, in which the *FMR1* gene harbors over 200 CGG repeats but is transcriptionally silent, the clinical features of FXTAS arise from a toxic gain of function of the elevated levels of *FMR1* transcript containing the long CGG tract. However, how this RNA leads to neuronal cell dysfunction is unknown. Here, we discuss the latest advances in the current understanding of the possible molecular basis of FXTAS.

## Review

### Introduction

Fragile X-associated tremor/ataxia syndrome (FXTAS) is a neurodegenerative disorder that affects older adults who have a large CGG-repeat tract in the 5′-untranslated region (UTR) of the *Fragile X Mental Retardation 1* (*FMR1*) gene [1]. Historically, carriers of Fragile X (FX) premutation alleles with 55 to 200 CGG repeats are considered at risk for FXTAS. The prevalence of premutation alleles is approximately 1 in 260 to 1 in 800 for males and 1 in 130- to 1 in 250 for females in the general population [2,3]. Given reduced penetrance of FXTAS, it is estimated that 1 in 2,000 men over the age of 50 years in the general population will show symptoms of FXTAS [2,4-6]. Clinical features of FXTAS include progressive intention tremor and gait ataxia, which is frequently accompanied by progressive cognitive decline, parkinsonism, peripheral neuropathy, and autonomic dysfunction [7]. The neuropathology of FXTAS consists of mild brain atrophy and degeneration of the cerebellum, including hyperintensity of the middle cerebellar peduncle (MCP), loss of Purkinje neuronal cells, spongiosis of the deep cerebellar white matter, Bergman gliosis, and swollen axons [8-10]. Immunocytochemical staining of post-mortem brain tissue from FXTAS reveals the presence of eosinophilic and ubiquitin-positive intranuclear inclusions that are broadly distributed throughout the brain, including in neurons and astrocytes [9,10], the spinal column, and several non-nervous tissues including thyroid, heart, and the Leydig cells in the testes [11,12].

In contrast to the absence of *FMR1* mRNA and protein expression seen in carriers of a full mutation (over 200 CGG repeats), individuals with premutation alleles have markedly increased expression of *FMR1* mRNA, but only moderately decreased FMRP levels [13-16]. FXTAS is not seen in carriers of fully silenced *FMR1* alleles, suggesting that a novel mechanism, involving increased expression of the long CGG repeat tract in the *FMR1* mRNA, is responsible for FXTAS. In support of this hypothesis, multiple studies have demonstrated adverse consequences of expressing CGG repeats in fly, mouse, and cell models [17-22]. Consistent with an RNA-based pathological mechanism, FXTAS has also been reported in individual carriers of intermediate alleles (45 to 55 CGG repeats) [23,24], and in full mutation allele carriers who are mosaics, both for repeat size and methylation, and who still express some *FMR1* mRNA [25-27]. In addition, there has been a report documenting the presence of intranuclear inclusions in the brains of three older adult males with Fragile X syndrome (FXS) [12]. These results have implications for the spectrum of FX-associated disorders, and suggest that the definition of FXTAS may need to be broadened to include individuals whose *FMR1* allele, irrespective of its size, makes sufficient RNA for its deleterious effects to be apparent.

How the RNA containing expanded CGG repeats leads to FXTAS pathogenesis is not yet fully known. This review will cover the recent advances in the understanding of the molecular mechanisms that may contribute to the pathogenesis of FXTAS, including the data presented at the First International Conference on *FMR1* Premutation (23 to 26 June, Perugia, Italy). For other aspects of FXTAS, there are a number of excellent reviews available [28-31], as well the additional articles published in this special issue of *JND*.

### CGG repeats are unstable, and tend to expand over time or with successive generations

Increased CGG repeat numbers are associated with an increased risk of FXTAS and with an increased severity and reduced age of onset of FXTAS symptoms [5,6]. The CGG repeat tract responsible for FXTAS is polymorphic in the human population. Normal alleles have between 6 and 45 repeats, and are relatively stable on intergenerational transmission. However, as the repeat number increases, so too does the likelihood that the repeat tract will expand or gain additional repeat units on intergenerational transfer. AGG interruptions, which are commonly seen within *FMR1* alleles, typically at 10 to 11 and 20 to 21 triplets from the 5′ end [32], are associated with a reduced risk of expansion [33,34]. Contractions do occur [35], but much less frequently. One of the consequences of the expansion bias is that alleles tend to increase in repeat number with successive generations. In addition to intergenerational expansion, somatic expansion is also seen in certain organs, including the brain in mice, and both lymphocytes and brain tissue in humans [27,36]. Somatic expansion may contribute to the repeat-length mosaicism that is seen in some human premutation carriers [27,37-40]. This somatic expansion has the potential to exacerbate FXTAS symptoms, particularly in carriers of alleles with more than 100 CGG repeats, where the repeat may be particularly prone to expansion.

The mechanism responsible for these expansions is unknown. A number of other diseases are known to result from expansions of tracts containing these repeats or other short repeat units. Whether or not these diseases, which are referred to collectively as the repeat expansion diseases [41], share a common expansion mechanism is unknown. However, the unusual nature of these mutations suggests that they might. The expansion bias clearly differentiates the instability in these diseases from the classic microsatellite instability seen in certain cancers, where the repeat is as likely to lose repeats as it is to gain them.

The individual strands of expanded CGG repeats, like other repeats that cause repeat expansion diseases, form secondary structures, including hairpins and quadruplexes [42]. These structures affect DNA processing enzymes such as DNA polymerase, both in vitro [42] and in vivo ([43,44]. It is generally thought that these structures are the trigger or substrate for expansion [41]. However, expansion in brain and liver, which are organs with a low proliferative capacity, along with expansion in mouse oocytes [45,46], suggest that the expansion mechanism may not involve aberrant DNA replication. Rather, given that oxidative stress exacerbates expansion in mice [47], it may be that expansion results from the aberrant repair of oxidized DNA or of DNA that is damaged in other ways. In contrast to generalized microsatellite instability, expansion in premutation mice actually requires *Msh2*[48]. However, whether expansion involves disruption of classic mismatch repair or involves another MSH2-dependent process is unknown. Although *Msh2* is required for expansion, it is not required for contractions [45,46,48]. Thus, it seems likely that expansions and contractions occur by different mechanisms, and the expansion bias seen in FX pedigrees may reflect a more efficient operation of the expansion process relative to the process that generates contractions.

### Expression of FMR1 mRNA is increased in premutation carriers

**Figure 1:**
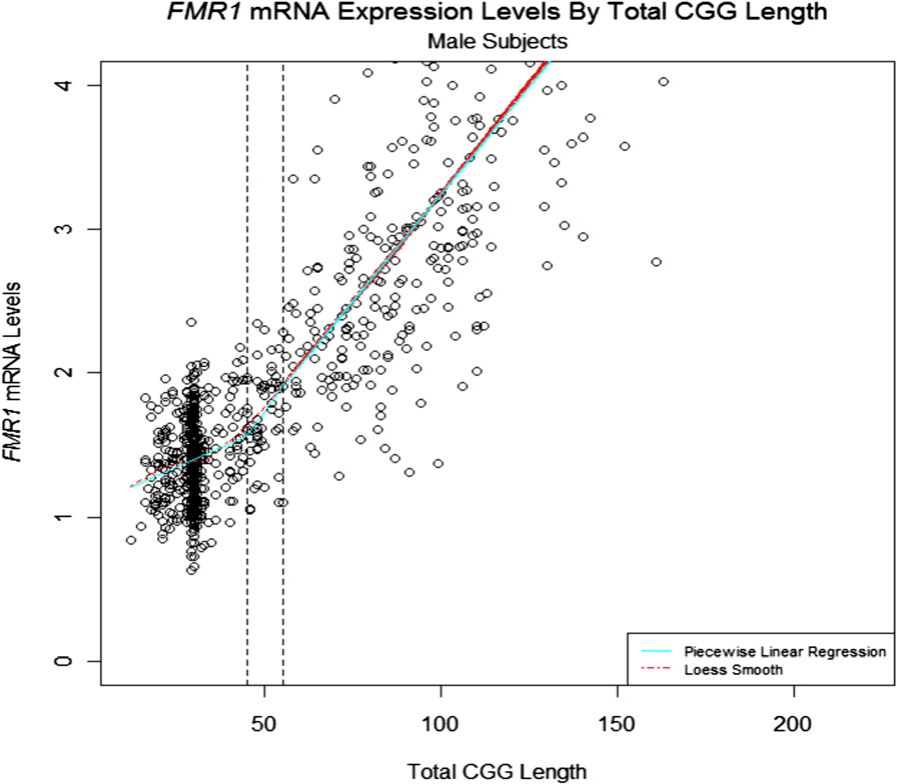
**Quantification of *FMR1 *mRNA levels in the three allele categories (normal, intermediate, and premutation) shows that *FMR1 *mRNA expression increases significantly with increasing CGG repeat number.** The solid blue line on the plot shows a piecewise linear regression fit, with *FMR1* mRNA expression increased significantly in all three groups (*P* = 0.012, *P* < 0.001, and *P* < 0.001 for normal, intermediate, and premutation carriers, respectively).

Several studies have shown that premutation alleles are characterized by high mRNA expression levels [13,15,16,49]. In our recent analysis, *FMR1* gene expression levels were measured in peripheral blood leukocytes from a total of 806 males across the whole range of CGG repeats, including normal individuals (n = 463), and individuals carrying intermediate (n = 60) and premutation (n = 283) alleles [27]. The results showed that *FMR1* mRNA levels increased with increased CGG repeat number, and that a significant increase (*P* < 0.001) was detectable for allele lengths as short as 35 CGG repeats (Figure 1). In addition, nuclear run-on experiments indicated that this elevated level of *FMR1* mRNA in premutation carriers is caused by increased transcription efficiency rather than increased mRNA stability [14,15]. Despite higher levels of *FMR1* transcripts, mild deficits of FMRP have been found in premutation carriers, and are probably due to a deficit in translational efficiency, particularly in the upper premutation range [13,16,49]. Thus, an FMRP deficiency is probably not the principal cause of FXTAS. Instead, the crucial observation that RNAs containing expanded CGG repeats accumulate in nuclear RNA aggregates in brain sections of patients with FXTAS [50] supports the notion that elevated levels of *FMR1* mRNA trigger neuronal toxicity. In support of this hypothesis, heterologous expression of 90 CGG repeats in *Drosophila melanogaster* was shown to cause neurodegeneration and formation of ubiquitin inclusions [18]. Similarly, a knock-in (KI) mouse model, in which the endogenous eight CGG repeats of the murine *Fmr1* were replaced with an expansion containing around 100 CGG repeats of human origin, showed ubiquitin-positive nuclear inclusions, and mild neuromotor and behavioral disturbances [17,51,52]. Finally, expression of transcripts containing 90 CGG repeats in a transgenic mouse model recapitulated some of the neuropathological and molecular features of FXTAS, despite the presence of a normal *Fmr1* allele [19] (see also review on animal models for FXTAS in this issue). These animal models show that the expression of *FMR1* mRNA containing expanded CGG repeats is both necessary and sufficient to cause pathological features characteristic of human FXTAS. Several mechanisms have been proposed to explain how increased expression of a RNA containing expanded CGG repeats could be pathogenic.

### Is pathology the result of an RNA gain-of-function mechanism?

The first recognized examples of RNA gain-of-function diseases were two other repeat expansion diseases, myotonic dystrophy type 1 and 2 (DM1 and DM2) [53]. DM is the most common muscular dystrophy in adults, and in this condition, RNAs containing hundreds to thousands of CUG (DM1) or CCUG (DM2) repeats accumulate in nuclear RNA aggregates that sequester the Muscleblind-like (MBNL) splicing factors. Depletion of the free pool of MBNL1 leads to specific alternative splicing changes, which ultimately result in the symptoms of DM [53]. Extending this RNA gain-of-function model to FXTAS, the expanded CGG repeats are predicted to sequester specific proteins, resulting in loss of their normal functions, which would ultimately cause the symptoms of FXTAS [54,55]. Consistent with this idea, Iwahashi and collaborators [56] identified more than 20 proteins from inclusions purified from brains of patients with FXTAS. Of these, two RNA binding proteins were of special interest. The first, hnRNP A2/B1 is mutated in families with inherited degeneration affecting muscle, brain, bone, and motor neurons [57], while the second, MBNL1, is the splicing factor that is involved in DM [58]. However, a role for MBNL1 in FXTAS has been excluded, because no genetic interaction between MBNL1 and CGG-mediated neurodegeneration was observed in the fly model of FXTAS [59], and no misregulation of splicing events regulated by MBNL1 was observed in brain samples from patients with FXTAS [60]. By contrast, binding of hnRNP A2/B1 to RNA containing expanded CGG repeats was confirmed by independent proteomic and in vitro analyses [60,61]. Furthermore, overexpression of hnRNP A2/B1 rescued the neurodegeneration in transgenic *Drosophila* expressing 90 CGG repeats [59,61]. Interaction of hnRNP A2/B1 with RNA containing expanded CGG repeats was evident in cytoplasmic cerebellar lysates. By contrast, nuclear hnRNP A2/B1 presented little binding to CGG RNA, suggesting that some modifications of hnRNP A2/B1, either in the nucleus or in the cytoplasm, may alter the ability of hnRNP A2/B1 to bind to CGG RNA repeats [59]. The importance of titration of the cytoplasmic pool of hnRNP A2/B1 was further demonstrated by expression of expanded CGG repeats in primary cultures of rat sympathetic neurons [62]. RNA containing CGG repeats competed for binding of hnRNP A2/B1 to BC1 RNA, a dendritic regulatory RNA, resulting in impaired dendritic delivery of the BC1 RNA [62]. However, no misregulation of splicing events regulated by nuclear hnRNP A2/B1 was observed in brain samples of patients with FXTAS [60]. Overall, these data suggest that expanded CGG repeats recruit hnRNP A2/B1, resulting in depletion of the cytoplasmic but not the nuclear pool of hnRNP A2/B1. In addition, the ability of hnRNP A proteins to unfold tetraplex RNA structures, formed by expanded CGG repeats [63,64], raises the possibility that hnRNP A2/B1 may also act as a RNA chaperone that destabilizes these RNA structures. Finally, Sofola and collaborators [59] demonstrated that hnRNP A2/B1 recruits, *in trans* and through protein-protein interactions, other proteins such as CUGBP1, an RNA binding protein, whose expression is increased in heart samples of patients with DM [65]. These data indicated that proteins binding to CGG RNA may recruit other proteins, resulting in dynamic aggregates that expand over time, a model later confirmed in COS7 cells expressing 60 CGG repeats [60]. Overexpression of either hnRNP A2/B1 or CUGBP1 rescued neurodegeneration in a *Drosophila* model of FXTAS, highlighting the potential importance of hnRNP A2/B1 and CUGBP1 to FXTAS pathology [59].

In addition to hnRNP A2/B1, proteomic analyses performed by Jin and collaborators [61] also showed that purine-rich binding protein α (Purα) binds robustly to RNA containing expanded CGG repeats. Purα is a single-stranded cytoplasmic DNA and RNA binding protein that has been implicated in many biological processes, including RNA transport and translation. Importantly, overexpression of Purα rescued neurodegeneration in a *Drosophila* model of FXTAS [61]. However, presence of Purα within nuclear aggregates in FXTAS brain samples is inconsistently observed. Jin et al. found Purα in cytoplasmic inclusions in *Drosophila* expressing 90 CGG repeats, and in inclusions in superior-mid temporal cortex neurons from human FXTAS brain sections [61]. By contrast, Iwashashi et al. did not detect Purα in purified inclusions from cerebral cortex of patients with FXTAS [56]. Furthermore, Purα-positive inclusions have not been observed in mouse models of FXTAS [66], or in hippocampal and cortical brain section of patients with FXTAS [67]. These results suggest that the composition of the inclusions varies from one brain region to the next and from one model organism to the other. Analogous to the recruitment of CUGBP1 by hnRNP A2/B1 to RNA containing expanded CGG repeats, Purα was shown to recruit Rm62, the *Drosophila* ortholog of the RNA helicase P68/DDX5 [68]. Expression of expanded CGG repeats resulted in the post-transcriptional downregulation of Rm62, ultimately resulting in nuclear accumulation of *Hsp70* mRNA and of other mRNAs involved in stress and immune responses [68]. Overexpression of Rm62 rescued neurodegeneration in flies expressing 90 CGG repeats, highlighting the potential importance of P68/DDX5 to FXTAS pathology [68].

SAM68, a splicing regulator encoded by the *KHDRBS1* gene, was also found in CGG RNA aggregates [60]. However, overexpression of SAM68 was not sufficient to rescue neuronal cell death induced by expression of expanded CGG repeats [67]. As with CUGBP1 and Rm62, SAM68 did not bind directly to CGG repeats, and recruitment of SAM68 within CGG RNA aggregates occurred in *trans* through protein-protein interactions [59]. Sellier and collaborators [67] also showed that DROSHA-DGCR8, the enzymatic complex that processes pri-microRNAs into pre-miRNAs, associated specifically with CGG repeats of pathogenic size. Sequestration of DROSHA-DGCR8 within CGG RNA aggregates resulted in reduced processing of pri-miRNAs in cells expressing expanded CGG repeats, and in brain samples from patients with FXTAS. Overexpression of DGCR8 rescued neuronal cell death induced by expression of expanded CGG repeats [67]. These results suggest that titration of DGCR8 by expanded CGG repeats is a leading event to CGG-induced neuronal cell death. However, recent analyses of miRNA expression in blood samples of patients with FXTAS and in *Drosophila* expressing CGG repeats did not show a global downregulation of miRNA, but rather, the expression of some specific miRNAs was misregulated [69,70]. Whether depletion of DROSHA-DGCR8 varies in blood and brain of patients with FXTAS, and whether the Drosha-Pasha complex is sequestered in cytoplasmic aggregates in *Drosophila* expressing expanded CGG repeats, remains to be determined. Similarly, whether overexpression of hnRNP A2/B1, P68/DDX5, DROSHA-DGCR8, or CUGBP1 rescues any phenotype in mouse models expressing expanded CGG repeats, would be necessary to determine the importance of these candidate proteins to FXTAS pathology.

**Figure 2:**
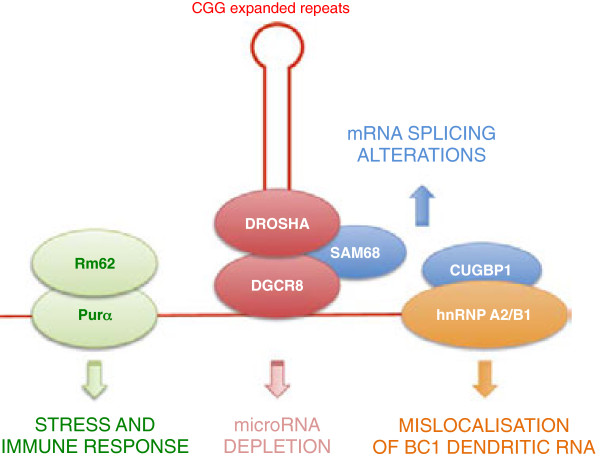
**Various RNA binding proteins have been found to associate with RNAs containing expanded CGG repeats.** Purα, DGCR8, and hnRNP A2/B1 bind directly to the CGG-containing RNA, whereas Rm62, SAM68, and CUGBP1 are recruited in *trans* through protein-protein interactions.

These caveats aside, the observations described above suggest that CGG repeats could be pathogenic by sequestering specific RNA binding proteins, resulting in loss of their normal functions, and thus lead to neuronal cell dysfunction (Figure 2) [56,58,61,67,68]. However, this attractive model has some weaknesses. First, the inclusions observed in FXTAS brain sections differ from those seen in DM, a typical RNA gain-of-function disorder. In FXTAS, inclusions are larger and ubiquitinated, and contain various chaperone proteins such as Hsp27, Hsp70, and αB-crystallin [9,56]. In short, these large inclusions resemble the aggregates seen in protein-mediated disorders, although they are negative for the typical proteins found in tauopathies, synucleinopathies, or polyQ disorders (for example, Huntington’s disease). Second, and most disconcerting, although inclusions in brain samples of patients with FXTAS contain the mutant *FMR1* RNA with expanded CGG repeats [50], a mouse model, in which the endogenous eight CGG repeats of *Fmr1* is replaced with an expansion containing around 100 CGG repeats, shows numerous ubiquitin inclusions but only rare aggregates of SAM68 or DROSHA-DGCR8, associated with rare RNA aggregates of expanded CGG repeats [60,67]. Similarly, overexpression of expanded CGG repeats leads to formation of nuclear RNA aggregate in some cell types, including primary cultures of hippocampal embryonic mouse neurons and PC12, COS7, and SKOV3 immortalized cell lines, but no RNA aggregates have been observed in A172, U-937, THP1, HeLa, HEK293, NG108-15, IMR-32, Neuro-2a, SH-SY5Y, SK-N-MC, or SK-N-SH cells [22,60]. In short, not all cell lines can support CGG repeat aggregate formation, whereas in DM, expression of expanded CUG or CCUG repeats consistently results in formation of RNA foci. Thirdly, a recent and provocative study demonstrates that the toxic effect of CGG repeats depends on their location [71]. Moving expanded CGG repeats from a 5′ UTR to a 3′ UTR position reduced their toxic effect in *Drosophila*, whereas expanded CUG or CCUG repeats were found to be pathogenic in whatever location tested, provided they were expressed in sufficient amounts to deplete MBNL proteins. These data led Todd and collaborators to reconsider the model of RNA binding protein sequestration, and to explore further the molecular mechanisms of FXTAS.

### Non-canonical AUG translation produces a polyglycine-containing protein in FXTAS

An unexpected observation, made by Todd and colleagues in flies transgenic for a construct containing 90 expanded CGG repeats cloned upstream of the green fluorescent protein (GFP) cDNA, was that some of the GFP signal was found in cytoplasmic inclusions. Western blotting analysis showed a band of the expected size for GFP, but also detected a protein 12 kDa larger [71]. Because translation of expanded CAG repeats in the absence of an ATG initiation codon (repeat-associated non-ATG translation or RAN translation) had been previously reported [72], Todd and collaborators tested whether expanded CGG repeats could be translated despite the fact that no ATG codon is present upstream of the repeats. Their analysis revealed that, indeed, translation of CGG repeats occurs in two out of the three frames, giving rise to short proteins containing either a polyalanine or a polyglycine stretch. Expression of the polyglycine protein resulted in the formation of protein inclusions, which were toxic both in neuronal transfected cells and in *Drosophila*. Further analyses of the polyglycine protein revealed that its translation was probably initiated at non-canonical AUG codons, such as CUG and GUG, which were located upstream of the CGG repeats. A role for non-canonical translation initiation in inclusion formation is consistent with data from two different KI mice mouse models. In a mouse model that showed numerous ubiquitin inclusions, the expanded CGG repeat from a human premutation allele was cloned, along with sequences upstream of the CGG repeats in humans that contained the non-AUG initiations codons [17]. By contrast, in a second mouse model, in which the mouse 5′ flanking sequence was retained, a stop codon was found to be located just upstream of the expanded CGG repeats [21]. These latter mice showed relatively few ubiquitinated aggregates, thus supporting the notion that non-ATG-initiated translation of the CGG tract is required to generate most of the inclusions [71]. That this unusual mode of translation may play a role in FXTAS is evidenced by the fact that, with the aid of specific antibodies, polyglycine protein can be seen in brain sections of patients with FXTAS [71]. Overall, these observations suggest that a protein gain of function may also occur in cells of patients with FXTAS. However, what contribution the polyglycine-containing or polyalanine-containing proteins make to the etiology of FXTAS is an exciting open question.

### Non-coding transcription of the FMR1 locus: a role in FMR1 mRNA toxicity?

The majority of the human genome is transcribed but not translated. Such RNAs are classified as long non-coding RNAs (lncRNAs) when longer than 200 nucleotides [73-75]. To date, relatively few lncRNAs have been functionally characterized, but increasing evidence suggests that many may have important functions, including the regulation of transcription, RNA processing and translation, DNA methylation, and chromatin architecture, both locally (*cis*-acting) and across some genomic distance (*trans*-acting) [76-78].

**Figure 3:**
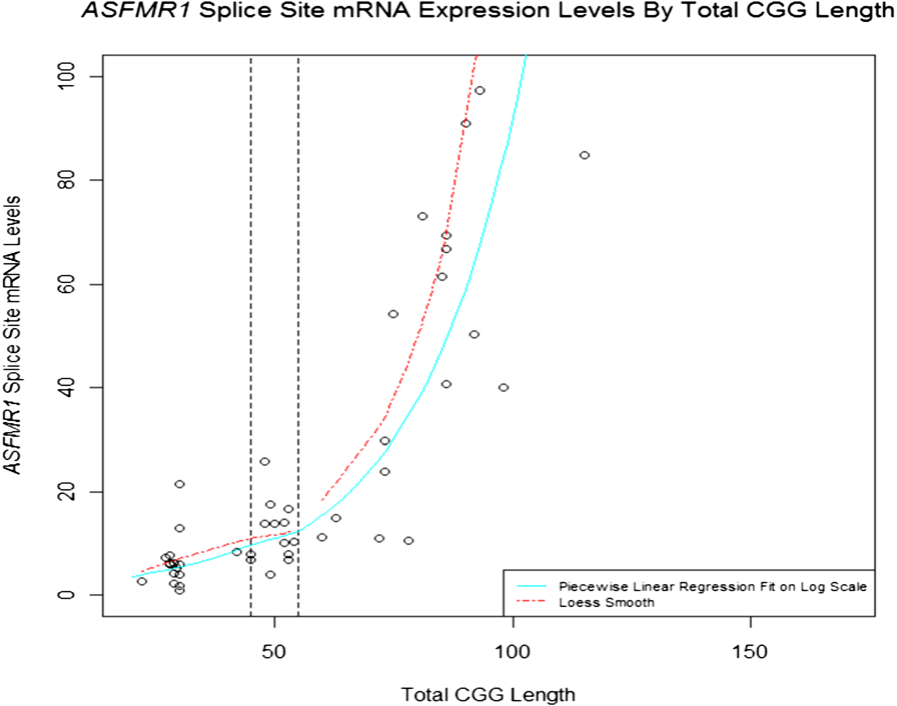
**Expression of the minor splice isoform in the *ASFMR1 *transcript (131 bp), located near the *ASFMR1 *promoter.** Expression of this isoform increases in premutation carriers (*P* < 0.001), and shows a similar trend in subjects with intermediate alleles (*P* = 0.0528) compared with normal alleles. The solid blue line on the plot shows a piecewise linear regression fit (fitted on the log scale then exponentiated for plotting), with separate slopes in the normal, intermediate, and premutation alleles.

In addition to the *FMR1* transcript, a variety of RNAs are produced from the *FMR1* locus. Therefore, it is possible that these lncRNAs produced from the *FMR1* locus may modulate certain aspects of FXS/FXTAS, as has been shown in other human diseases [79]. For example, Kumari and Usdin described an abundant antisense transcript of about 5 kb that spans the region upstream of the FMR1 promoter, and whose expression does not change in response to repeat expansion [80]. By contrast Ladd and coworkers described a transcript, Antisense *FMR1 (ASFMR1*), that spans the expanded CGG repeats and whose expression is elevated in lymphoblastoid cells and peripheral blood leukocytes of individuals with premutation alleles, while it is not expressed in those with full mutation alleles [81]. Multiple splice forms of *ASFMR1* have been identified, which show differential expression in carriers of premutation and normal alleles [81]. One of these *ASFMR1* splice variants contains a small intron that uses a non-consensus CT-AC splice site that is transcribed in a premutation cell line, but is absent in a normal cell line [81]. We compared the expression levels of this *ASFMR1* isoform in blood from individuals with alleles ranging from normal to premutation, and found a significant increase with CGG repeat number (*P* < 0.001) (Figure 3) [27]. Of interest, both unspliced and spliced *ASFMR1* transcripts contain putative open-reading frames encoding polyproline peptides, resulting from antisense-oriented translation of the expanded CGG repeats [81]. Whether *ASFMR1* containing expanded CCG repeats is translated and participates in the formation of the pathogenic nuclear inclusions observed in patients with FXTAS remain to be tested.

Another antisense transcript, *FMR4*, originates upstream of the *FMR1* start site, and covers 2.4 kb of sequence [82]. *FMR4* is widely expressed in fetal and adult human tissues, and throughout human and macaque brain regions. Expression of *FMR4*, like that of *ASFMR1* and *FMR1*, is increased in brain tissue of premutation individuals and is silenced in individuals with the full mutation [82]. Importantly, *FMR4* overexpression was shown to increase cell proliferation, whereas *FMR4* downregulation induced apoptosis in vitro [82]. Additionally, no *cis*-acting effect was observed upon expression of the *FMR1* gene. Therefore, it was hypothesized that *FMR4* influences proliferation pathways in *trans*, by targeting distal genomic loci. Current work is focused on defining a role for this transcript, as it has been found to affect the chromatin state and transcription of several genes involved in neuronal differentiation, axon guidance, and synaptic signaling, as well as cell cycle regulators (Peschansky and Pastori, unpublished data).

Two new transcripts arising from the *FMR1* locus, *FMR5* and *FMR6,* were recently identified [83]. *FMR5* is a sense-oriented lncRNA transcribed from approximately 1 kb upstream of the *FMR1* transcription start site (TSS). *FMR5* is not differentially expressed in human brain from unaffected individuals compared with full mutation and premutation patients, suggesting that its transcription is independent of CGG repeat expansion. Furthermore, the TSS of *FMR5* appears not to be affected by the chromatin silencing that occurs within full mutation alleles, or by the open chromatin hypothesized to increase transcription of *FMR1* premutation alleles. *FMR6* is a spliced long antisense transcript, 600 nucleotides i, whose sequence is entirely complementary to the 3′ region of *FMR1*[83]. It begins in the 3′UTR, ends in exon 15 of *FMR1*, and uses the same splice junctions as *FMR1*. An unexpected finding was that *FMR6* is reduced in premutation carriers, suggesting that abnormal transcription and/or chromatin remodeling occurs toward the distal end of the locus. However, the chromatin marks associated with the 3′ end of *FMR1* in premutation carriers have yet to be described. The function of *FMR6* remains to be identified, but its complementarity to the 3′ region of *FMR1* presents several interesting possibilities. *FMR6* may bind to *FMR1* mRNA, thereby regulating the stability, splicing, subcellular localization, or translational efficiency of *FMR1*, as has been described for other lncRNAs [77]. Notably, *FMR6* overlaps miR-19a and miR-19b binding sites in the *FMR1* 3′ UTR [84], suggesting that *FMR6* may modulate the stability or translational efficiency of *FMR1* by interfering with microRNA binding. Overall, these results highlight the importance of non-coding transcription of the *FMR1* locus. However, much work remains to fully understand the relevance of these transcripts to the pathology observed in premutation carriers.

## Conclusion

The restriction of FXTAS clinical features to unmethylated, transcriptionally active alleles with large CGG repeat numbers suggests that the expression of a mutant RNA is pathogenic to neuronal cells [55]. This hypothesis is supported by data from cell, fly, and mouse models [17-22]. However, how these RNAs cause neuronal cell dysfunction and FXTAS symptoms remains unclear. One model proposes that the RNA containing expanded CGG repeats is pathogenic via its sequestration of specific RNA binding proteins. Various proteins, including Purα, Rm62, CUGBP1, hnRNP A2/B1, SAM68, and DROSHA-DGCR8, have been shown to bind directly or through a protein partner to expanded CGG repeats [56,59,61,67,68]. However, it remains to be tested whether overexpression of these candidate proteins rescues any phenotype in mouse models expressing expanded CGG repeats. A second mechanism involves non-canonical translation initiation of expanded CGG repeats. resulting in expression of toxic polyglycine-containing and polyalanine-containing proteins [71]; however, how these proteins promote neuronal cell dysfunction is an open question. A third model is associated with the expression of antisense *FMR1* transcripts. Further investigation is needed to evaluate the pathological consequences of expression of *ASFMR1* or other long non-coding RNA mapping within the *FMR1* gene. Finally, although decreased expression of FMRP is probably not the principal cause of FXTAS, it cannot be excluded that a reduction in FMRP plays a role in modulating some of FXTAS features. In that context, the level of FMRP depletion in brain samples from a larger cohort of patients with FXTAS needs to be measured.

**Figure 4:**
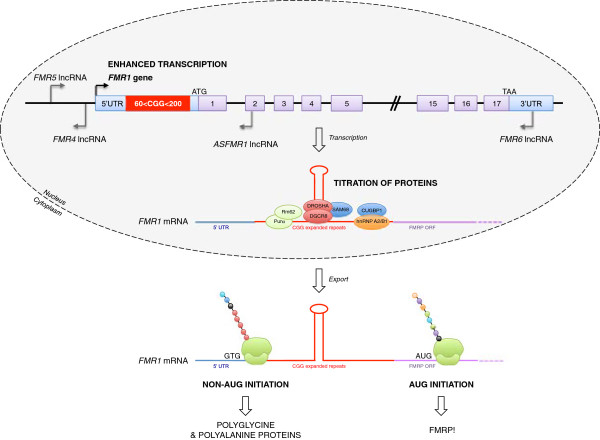
**Mechanisms that may contribute to fragile X-associated tremor/ataxia (FXTAS) pathology.** Expression of *FMR1* mRNA and associated antisense transcripts are increased inpremutation carriers. Some transcripts accumulate in the nucleus and recruit various RNA binding proteins, while export and non-AUG translation results in production of polyglycine-containing or polyalanine-containing proteins.

In conclusion, in addition to increased *FMR1* mRNA production, protein titration, non-AUG translation, antisense transcription, and decreased expression of FMRP are a number of non-exclusive mechanisms that may all contribute to FXTAS pathology. It is possible that contributions to pathology from more than one mechanism may help to explain the great variability in clinical presentation of premutation individuals, aspects of which have heretofore not been accounted for by CGG expansion size, mosaicism, methylation, alternative spliced isoforms, additional genomic changes, or other known factors. Thus, more work is needed to determine the relative contribution of these processes to disease pathology in this multifaceted disorder (Figure 4).

## Competing interests

The authors declare that they have no competing interests.

## Authors’ contributions

CS, KU, CP, VJP, FT and NCB wrote the paper. All authors read and approved the final manuscript.

## References

[B1] Hagerman RJ Leehey M Heinrichs W Tassone F Wilson R Hills J Grigsby J Gage B Hagerman PJ Intention tremor, parkinsonism, and generalized brain atrophy in male carriers of fragile X Neurology 2001 57 127 130 11445641 10.1212/wnl.57.1.127

[B2] Tassone F Iong KP Tong TH Lo J Gane LW Berry-Kravis E Nguyen D Mu LY Laffin J Bailey DB Hagerman RJ FMR1 CGG allele size and prevalence ascertained through newborn screening in the United States Genome Med 2012 4 100 23259642 10.1186/gm401 PMC4064316

[B3] Maenner MJ Baker MW Broman KW Tian J Barnes JK Atkins A McPherson E Hong J Brilliant MH Mailick MR FMR1 CGG expansions: prevalence and sex ratios Am J Med Genet B Neuropsychiatr Genet 2013 162B 466 473 23740716 10.1002/ajmg.b.32176 PMC3885228

[B4] Jacquemont S Hagerman RJ Leehey MA Hall DA Levine RA Brunberg JA Zhang L Jardini T Gane LW Harris SW Herman K Grigsby J Greco CM Berry-Kravis E Tassone F Hagerman PJ Penetrance of the fragile X-associated tremor/ataxia syndrome in a premutation carrier population Jama 2004 291 460 469 14747503 10.1001/jama.291.4.460

[B5] Tassone F Adams J Berry-Kravis EM Cohen SS Brusco A Leehey MA Li L Hagerman RJ Hagerman PJ CGG repeat length correlates with age of onset of motor signs of the fragile X-associated tremor/ataxia syndrome (FXTAS) Am J Med Genet B Neuropsychiatr Genet 2007 144B 566 569 17427188 10.1002/ajmg.b.30482

[B6] Leehey MA Berry-Kravis E Goetz CG Zhang L Hall DA Li L Rice CD Lara R Cogswell J Reynolds A Gane L Jacquemont S Tassone F Grigsby J Hagerman RJ Hagerman PJ FMR1 CGG repeat length predicts motor dysfunction in premutation carriers Neurology 2008 70 1397 1402 18057320 10.1212/01.wnl.0000281692.98200.f5 PMC2685188

[B7] Jacquemont S Hagerman RJ Leehey M Grigsby J Zhang L Brunberg JA Greco C Des Portes V Jardini T Levine R Berry-Kravis E Brown WT Schaeffer S Kissel J Tassone F Hagerman PJ Fragile X premutation tremor/ataxia syndrome: molecular, clinical, and neuroimaging correlates Am J Hum Genet 2003 72 869 878 12638084 10.1086/374321 PMC1180350

[B8] Brunberg JA Jacquemont S Hagerman RJ Berry-Kravis EM Grigsby J Leehey MA Tassone F Brown WT Greco CM Hagerman PJ Fragile X premutation carriers: characteristic MR imaging findings of adult male patients with progressive cerebellar and cognitive dysfunction AJNR Am J Neuroradiol 2002 23 1757 1766 12427636 PMC8185834

[B9] Greco CM Hagerman RJ Tassone F Chudley AE Del Bigio MR Jacquemont S Leehey M Hagerman PJ Neuronal intranuclear inclusions in a new cerebellar tremor/ataxia syndrome among fragile X carriers Brain 2002 125 1760 1771 12135967 10.1093/brain/awf184

[B10] Greco CM Berman RF Martin RM Tassone F Schwartz PH Chang A Trapp BD Iwahashi C Brunberg J Grigsby J Hessl D Becker EJ Papazian J Leehey MA Hagerman RJ Hagerman PJ Neuropathology of fragile X-associated tremor/ataxia syndrome (FXTAS) Brain 2006 129 243 255 16332642 10.1093/brain/awh683

[B11] Greco CM Soontrapornchai K Wirojanan J Gould JE Hagerman PJ Hagerman RJ Testicular and pituitary inclusion formation in fragile X associated tremor/ataxia syndrome J Urol 2007 177 1434 1437 17382748 10.1016/j.juro.2006.11.097

[B12] Hunsaker MR Greco CM Spath MA Smits AP Navarro CS Tassone F Kros JM Severijnen LA Berry-Kravis EM Berman RF Hagerman PJ Willemsen R Hagerman RJ Hukema RK Widespread non-central nervous system organ pathology in fragile X premutation carriers with fragile X-associated tremor/ataxia syndrome and CGG knock-in mice Acta Neuropathol 2011 122 467 479 21785977 10.1007/s00401-011-0860-9 PMC3222079

[B13] Kenneson A Zhang F Hagedorn CH Warren ST Reduced FMRP and increased FMR1 transcription is proportionally associated with CGG repeat number in intermediate-length and premutation carriers Hum Mol Genet 2001 10 1449 1454 11448936 10.1093/hmg/10.14.1449

[B14] Tassone F Beilina A Carosi C Albertosi S Bagni C Li L Glover K Bentley D Hagerman PJ Elevated FMR1 mRNA in premutation carriers is due to increased transcription RNA 2007 13 555 562 17283214 10.1261/rna.280807 PMC1831862

[B15] Tassone F Hagerman RJ Taylor AK Gane LW Godfrey TE Hagerman PJ Elevated levels of FMR1 mRNA in carrier males: a new mechanism of involvement in the fragile-X syndrome Am J Hum Genet 2000 66 6 15 10631132 10.1086/302720 PMC1288349

[B16] Peprah E He W Allen E Oliver T Boyne A Sherman SL Examination of FMR1 transcript and protein levels among 74 premutation carriers J Hum Genet 2010 55 66 68 19927162 10.1038/jhg.2009.121 PMC4122982

[B17] Willemsen R Hoogeveen-Westerveld M Reis S Holstege J Severijnen LA Nieuwenhuizen IM Schrier M van Unen L Tassone F Hoogeveen AT Hagerman PJ Mientjes EJ Oostra BA The FMR1 CGG repeat mouse displays ubiquitin-positive intranuclear neuronal inclusions; implications for the cerebellar tremor/ataxia syndrome Hum Mol Genet 2003 12 949 959 12700164 10.1093/hmg/ddg114

[B18] Jin P Zarnescu DC Zhang F Pearson CE Lucchesi JC Moses K Warren ST RNA-mediated neurodegeneration caused by the fragile X premutation rCGG repeats in Drosophila Neuron 2003 39 739 747 12948442 10.1016/s0896-6273(03)00533-6

[B19] Hashem V Galloway JN Mori M Willemsen R Oostra BA Paylor R Nelson DL Ectopic expression of CGG containing mRNA is neurotoxic in mammals Hum Mol Genet 2009 18 2443 2451 19377084 10.1093/hmg/ddp182 PMC2694692

[B20] Handa V Goldwater D Stiles D Cam M Poy G Kumari D Usdin K Long CGG-repeat tracts are toxic to human cells: implications for carriers of Fragile X premutation alleles FEBS Lett 2005 579 2702 2708 15862312 10.1016/j.febslet.2005.04.004

[B21] Entezam A Biacsi R Orrison B Saha T Hoffman GE Grabczyk E Nussbaum RL Usdin K Regional FMRP deficits and large repeat expansions into the full mutation range in a new Fragile X premutation mouse model Gene 2007 395 125 134 17442505 10.1016/j.gene.2007.02.026 PMC1950257

[B22] Arocena DG Iwahashi CK Won N Beilina A Ludwig AL Tassone F Schwartz PH Hagerman PJ Induction of inclusion formation and disruption of lamin A/C structure by premutation CGG-repeat RNA in human cultured neural cells Hum Mol Genet 2005 14 3661 3671 16239243 10.1093/hmg/ddi394

[B23] Hall D Tassone F Klepitskaya O Leehey M Fragile X-associated tremor ataxia syndrome in FMR1 gray zone allele carriers Mov Disord 2012 27 296 300 22161987 10.1002/mds.24021 PMC4286243

[B24] Liu Y Winarni TI Zhang L Tassone F Hagerman RJ Fragile X-associated tremor/ataxia syndrome (FXTAS) in grey zone carriers Clin Genet 2013 84 74 77 23009394 10.1111/cge.12026 PMC4991824

[B25] Loesch DZ Sherwell S Kinsella G Tassone F Taylor A Amor D Sung S Evans A Fragile X-associated tremor/ataxia phenotype in a male carrier of unmethylated full mutation in the FMR1 gene Clin Genet 2012 82 88 92 21476992 10.1111/j.1399-0004.2011.01675.x

[B26] Santa María L Pugin A Alliende M Aliaga S Curotto B Aravena T Tang HT Mendoza-Morales G Hagerman R Tassone F Fxtas in an unmethylated mosaic male with fragile X syndrome from chile Clin Genet 2013 doi: 10.1111/cge.12278. [Epub ahead of print] 10.1111/cge.12278 PMC4004716 24028275

[B27] Pretto DI Tang HT Lo J Morales G Hagerman R Hagerman PJ Tassone F CGG-repeat number and methylation instability in premutation alleles 2013 Perugia, Italy: First International Conference on FMR1 Premutation 23 to 26 June 2013

[B28] Willemsen R Levenga J Oostra BA CGG repeat in the FMR1 gene: size matters Clin Genet 2011 80 214 225 21651511 10.1111/j.1399-0004.2011.01723.x PMC3151325

[B29] Li Y Jin P RNA-mediated neurodegeneration in fragile X-associated tremor/ataxia syndrome Brain Res 2012 1462 112 117 22459047 10.1016/j.brainres.2012.02.057 PMC3372578

[B30] Hagerman R Hagerman P Advances in clinical and molecular understanding of the FMR1 premutation and fragile X-associated tremor/ataxia syndrome Lancet Neurol 2013 12 786 798 23867198 10.1016/S1474-4422(13)70125-X PMC3922535

[B31] Garcia-Arocena D Hagerman PJ Advances in understanding the molecular basis of FXTAS Hum Mol Genet 2010 19 R83 R89 20430935 10.1093/hmg/ddq166 PMC2875053

[B32] Eichler EE Holden JJ Popovich BW Reiss AL Snow K Thibodeau SN Richards CS Ward PA Nelson DL Length of uninterrupted CGG repeats determines instability in the FMR1 gene Nat Genet 1994 8 88 94 7987398 10.1038/ng0994-88

[B33] Nolin SL Sah S Glicksman A Sherman SL Allen E Berry-Kravis E Tassone F Yrigollen C Cronister A Jodah M Ersalesi N Dobkin C Brown WT Shroff R Latham GJ Hadd AG Fragile X AGG analysis provides new risk predictions for 45–69 repeat alleles Am J Med Genet A 2013 161 771 778 23444167 10.1002/ajmg.a.35833 PMC4396070

[B34] Yrigollen CM Durbin-Johnson B Gane L Nelson DL Hagerman R Hagerman PJ Tassone F AGG interruptions within the maternal FMR1 gene reduce the risk of offspring with fragile X syndrome Genet Med 2012 14 729 736 22498846 10.1038/gim.2012.34 PMC3990283

[B35] Tabolacci E Pomponi MG Pietrobono R Chiurazzi P Neri G A unique case of reversion to normal size of a maternal premutation FMR1 allele in a normal boy Eur J Hum Genet 2008 16 209 214 17971832 10.1038/sj.ejhg.5201949

[B36] Lokanga RA Entezam A Kumari D Yudkin D Qin M Smith CB Usdin K Somatic expansion in mouse and human carriers of fragile X premutation alleles Hum Mutat 2013 34 157 166 22887750 10.1002/humu.22177 PMC3524353

[B37] Grasso M Faravelli F Lo Nigro C Chiurazzi P Sperandeo MP Argusti A Pomponi MG Lecora M Sebastio GF Perroni L Andria G Neri G Bricarelli FD Mosaicism for the full mutation and a microdeletion involving the CGG repeat and flanking sequences in the FMR1 gene in eight fragile X patients Am J Med Genet 1999 85 311 316 10398249 10.1002/(sici)1096-8628(19990730)85:3<311::aid-ajmg24>3.0.co;2-a

[B38] Govaerts LC Smit AE Saris JJ VanderWerf F Willemsen R Bakker CE De Zeeuw CI Oostra BA Exceptional good cognitive and phenotypic profile in a male carrying a mosaic mutation in the FMR1 gene Clin Genet 2007 72 138 144 17661818 10.1111/j.1399-0004.2007.00829.x

[B39] Hantash FM Goos DG Tsao D Quan F Buller-Burckle A Peng M Jarvis M Sun W Strom CM Qualitative assessment of FMR1 (CGG)n triplet repeat status in normal, intermediate, premutation, full mutation, and mosaic carriers in both sexes: implications for fragile X syndrome carrier and newborn screening Genet Med 2010 12 162 173 20168238 10.1097/GIM.0b013e3181d0d40e

[B40] Nolin SL Glicksman A Houck GE Jr Brown WT Dobkin CS Mosaicism in fragile X affected males Am J Med Genet 1994 51 509 512 7943031 10.1002/ajmg.1320510444

[B41] Mirkin SM Expandable DNA repeats and human disease Nature 2007 447 932 940 17581576 10.1038/nature05977

[B42] Usdin K Woodford KJ CGG repeats associated with DNA instability and chromosome fragility form structures that block DNA synthesis in vitro Nucleic Acids Res 1995 23 4202 4209 7479085 10.1093/nar/23.20.4202 PMC307363

[B43] Voineagu I Surka CF Shishkin AA Krasilnikova MM Mirkin SM Replisome stalling and stabilization at CGG repeats, which are responsible for chromosomal fragility Nat Struct Mol Biol 2009 16 226 228 19136957 10.1038/nsmb.1527 PMC2837601

[B44] Gerhardt J Tomishima MJ Zaninovic N Colak D Yan Z Zhan Q Rosenwaks Z Jaffrey SR Schildkraut CL The DNA replication program is altered at the FMR1 locus in fragile X embryonic stem cells 2013 Perugia, Italy: First International Conference on FMR1 Premutation 23 to 26 June 2013 10.1016/j.molcel.2013.10.029 PMC3920742 24289922

[B45] Entezam A Usdin K ATR protects the genome against CGG. CCG-repeat expansion in Fragile X premutation mice Nucleic Acids Res 2008 36 1050 1056 18160412 10.1093/nar/gkm1136 PMC2241920

[B46] Entezam A Usdin K ATM and ATR protect the genome against two different types of tandem repeat instability in Fragile X premutation mice Nucleic Acids Res 2009 37 6371 6377 19710035 10.1093/nar/gkp666 PMC2770655

[B47] Entezam A Lokanga AR Le W Hoffman G Usdin K Potassium bromate, a potent DNA oxidizing agent, exacerbates germline repeat expansion in a fragile X premutation mouse model Hum Mutat 2010 31 611 616 20213777 10.1002/humu.21237 PMC2951473

[B48] Lokanga RA Zhao X-N Usdin K The mismatch repair protein MSH2 is rate-limiting for repeat expansion in a Fragile X premutation mouse model Hum Mutat 2013 doi: 10.1002/humu.22464. [Epub ahead of print] 10.1002/humu.22464 PMC3951054 24130133

[B49] Allen EG Sherman S Abramowitz A Leslie M Novak G Rusin M Scott E Letz R Examination of the effect of the polymorphic CGG repeat in the FMR1 gene on cognitive performance Behav Genet 2005 35 435 445 15971024 10.1007/s10519-005-2792-4

[B50] Tassone F Iwahashi C Hagerman PJ FMR1 RNA within the intranuclear inclusions of fragile X-associated tremor/ataxia syndrome (FXTAS) RNA Biol 2004 1 103 105 17179750 10.4161/rna.1.2.1035

[B51] Brouwer JR Huizer K Severijnen LA Hukema RK Berman RF Oostra BA Willemsen R CGG-repeat length and neuropathological and molecular correlates in a mouse model for fragile X-associated tremor/ataxia syndrome J Neurochem 2008 107 1671 1682 19014369 10.1111/j.1471-4159.2008.05747.x PMC2605773

[B52] Van Dam D Errijgers V Kooy RF Willemsen R Mientjes E Oostra BA De Deyn PP Cognitive decline, neuromotor and behavioural disturbances in a mouse model for fragile-X-associated tremor/ataxia syndrome (FXTAS) Behav Brain Res 2005 162 233 239 15876460 10.1016/j.bbr.2005.03.007

[B53] Ranum LP Cooper TA RNA-mediated neuromuscular disorders Annu Rev Neurosci 2006 29 259 277 16776586 10.1146/annurev.neuro.29.051605.113014

[B54] Nelson DL Orr HT Warren ST The unstable repeats–three evolving faces of neurological disease Neuron 2013 77 825 843 23473314 10.1016/j.neuron.2013.02.022 PMC3608403

[B55] Hagerman PJ Hagerman RJ The fragile-X premutation: a maturing perspective Am J Hum Genet 2004 74 805 816 15052536 10.1086/386296 PMC1181976

[B56] Iwahashi CK Yasui DH An HJ Greco CM Tassone F Nannen K Babineau B Lebrilla CB Hagerman RJ Hagerman PJ Protein composition of the intranuclear inclusions of FXTAS Brain 2006 129 256 271 16246864 10.1093/brain/awh650

[B57] Kim HJ Kim NC Wang YD Scarborough EA Moore J Diaz Z MacLea KS Freibaum B Li S Molliex A Kanagaraj AP Carter R Boylan KB Wojtas AM Rademakers R Pinkus JL Greenberg SA Trojanowski JQ Traynor BJ Smith BN Topp S Gkazi AS Miller J Shaw CE Kottlors M Kirschner J Pestronk A Li YR Ford AF Gitler AD Mutations in prion-like domains in hnRNPA2B1 and hnRNPA1 cause multisystem proteinopathy and ALS Nature 2013 495 467 473 23455423 10.1038/nature11922 PMC3756911

[B58] Kanadia RN Johnstone KA Mankodi A Lungu C Thornton CA Esson D Timmers AM Hauswirth WW Swanson MS A muscleblind knockout model for myotonic dystrophy Science 2003 302 1978 1980 14671308 10.1126/science.1088583

[B59] Sofola OA Jin P Qin Y Duan R Liu H de Haro M Nelson DL Botas J RNA-binding proteins hnRNP A2/B1 and CUGBP1 suppress fragile X CGG premutation repeat-induced neurodegeneration in a Drosophila model of FXTAS Neuron 2007 55 565 571 17698010 10.1016/j.neuron.2007.07.021 PMC2215388

[B60] Sellier C Rau F Liu Y Tassone F Hukema RK Gattoni R Schneider A Richard S Willemsen R Elliott DJ Hagerman PJ Charlet-Berguerand N Sam68 sequestration and partial loss of function are associated with splicing alterations in FXTAS patients Embo J 2010 29 1248 1261 20186122 10.1038/emboj.2010.21 PMC2857464

[B61] Jin P Duan R Qurashi A Qin Y Tian D Rosser TC Liu H Feng Y Warren ST Pur alpha binds to rCGG repeats and modulates repeat-mediated neurodegeneration in a Drosophila model of fragile X tremor/ataxia syndrome Neuron 2007 55 556 564 17698009 10.1016/j.neuron.2007.07.020 PMC1994817

[B62] Muslimov IA Patel MV Rose A Tiedge H Spatial code recognition in neuronal RNA targeting: role of RNA-hnRNP A2 interactions J Cell Biol 2011 194 441 457 21807882 10.1083/jcb.201010027 PMC3153643

[B63] Khateb S Weisman-Shomer P Hershco I Loeb LA Fry M Destabilization of tetraplex structures of the fragile X repeat sequence (CGG)n is mediated by homolog-conserved domains in three members of the hnRNP family Nucleic Acids Res 2004 32 4145 4154 15302914 10.1093/nar/gkh745 PMC514371

[B64] Ofer N Weisman-Shomer P Shklover J Fry M The quadruplex r(CGG)n destabilizing cationic porphyrin TMPyP4 cooperates with hnRNPs to increase the translation efficiency of fragile X premutation mRNA Nucleic Acids Res 2009 37 2712 2722 19273535 10.1093/nar/gkp130 PMC2677883

[B65] Kuyumcu-Martinez NM Wang GS Cooper TA Increased steady-state levels of CUGBP1 in myotonic dystrophy 1 are due to PKC-mediated hyperphosphorylation Mol Cell 2007 28 68 78 17936705 10.1016/j.molcel.2007.07.027 PMC2083558

[B66] Galloway JN Nelson DL Evidence for RNA-mediated toxicity in the fragile X-associated tremor/ataxia syndrome Future Neurol 2009 4 785 20161676 10.2217/fnl.09.44 PMC2821051

[B67] Sellier C Freyermuth F Tabet R Tran T He F Ruffenach F Alunni V Moine H Thibault C Page A Tassone F Willemsen R Disney MD Hagerman PJ Todd PK Charlet-Berguerand N Sequestration of DROSHA and DGCR8 by expanded CGG RNA repeats alters microRNA processing in fragile X-associated tremor/ataxia syndrome Cell Rep 2013 3 869 880 23478018 10.1016/j.celrep.2013.02.004 PMC3639429

[B68] Qurashi A Li W Zhou JY Peng J Jin P Nuclear accumulation of stress response mRNAs contributes to the neurodegeneration caused by Fragile X premutation rCGG repeats PLoS Genet 2011 7 e1002102 21655086 10.1371/journal.pgen.1002102 PMC3107199

[B69] Alvarez-Mora MI Rodriguez-Revenga L Madrigal I Torres-Silva F Mateu-Huertas E Lizano E Friedländer MR Martí E Estivill X Milà M MicroRNA expression profiling in blood from fragile X-associated tremor/ataxia syndrome patients Genes Brain Behav 2013 12 595 603 23790110 10.1111/gbb.12061

[B70] Tan H Poidevin M Li H Chen D Jin P MicroRNA-277 modulates the neurodegeneration caused by Fragile X premutation rCGG repeats PLoS Genet 2012 8 e1002681 22570635 10.1371/journal.pgen.1002681 PMC3343002

[B71] Todd PK Oh SY Krans A He F Sellier C Frazer M Renoux AJ Chen KC Scaglione KM Basrur V Elenitoba-Johnson K Vonsattel JP Louis ED Sutton MA Taylor JP Mills RE Charlet-Berguerand N Paulson HL CGG repeat-associated translation mediates neurodegeneration in fragile X tremor ataxia syndrome Neuron 2013 78 440 455 23602499 10.1016/j.neuron.2013.03.026 PMC3831531

[B72] Zu T Gibbens B Doty NS Gomes-Pereira M Huguet A Stone MD Margolis J Peterson M Markowski TW Ingram MA Nan Z Forster C Low WC Schoser B Somia NV Clark HB Schmechel S Bitterman PB Gourdon G Swanson MS Moseley M Ranum LP Non-ATG-initiated translation directed by microsatellite expansions Proc Natl Acad Sci U S A 2011 108 260 265 21173221 10.1073/pnas.1013343108 PMC3017129

[B73] Mattick JS Makunin IV Small regulatory RNAs in mammals Hum Mol Genet 2005 14 1 R121 R132 15809264 10.1093/hmg/ddi101

[B74] Djebali S Davis CA Merkel A Dobin A Lassmann T Mortazavi A Tanzer A Lagarde J Lin W Schlesinger F Xue C Marinov GK Khatun J Williams BA Zaleski C Rozowsky J Röder M Kokocinski F Abdelhamid RF Alioto T Antoshechkin I Baer MT Bar NS Batut P Bell K Bell I Chakrabortty S Chen X Chrast J Curado J Landscape of transcription in human cells Nature 2012 489 101 108 22955620 10.1038/nature11233 PMC3684276

[B75] Yan B Wang Z Long noncoding RNA: its physiological and pathological roles DNA Cell Biol 2012 31 Suppl 1 S34 S41 22612272 10.1089/dna.2011.1544

[B76] Lee JT Epigenetic regulation by long noncoding RNAs Science 2012 338 1435 1439 23239728 10.1126/science.1231776

[B77] Faghihi MA Wahlestedt C Regulatory roles of natural antisense transcripts Nat Rev Mol Cell Biol 2009 10 637 643 19638999 10.1038/nrm2738 PMC2850559

[B78] Magistri M Faghihi MA St Laurent G 3rd, Wahlestedt C: Regulation of chromatin structure by long noncoding RNAs: focus on natural antisense transcripts Trends Genet 2012 28 389 396 22541732 10.1016/j.tig.2012.03.013 PMC3768148

[B79] Pastori C Wahlestedt C Involvement of long noncoding RNAs in diseases affecting the central nervous system RNA Biol 2012 9 860 870 22699553 10.4161/rna.20482 PMC3495748

[B80] Kumari D Usdin K The distribution of repressive histone modifications on silenced FMR1 alleles provides clues to the mechanism of gene silencing in fragile X syndrome Hum Mol Genet 2010 19 4634 4642 20843831 10.1093/hmg/ddq394 PMC2972696

[B81] Ladd PD Smith LE Rabaia NA Moore JM Georges SA Hansen RS Hagerman RJ Tassone F Tapscott SJ Filippova GN An antisense transcript spanning the CGG repeat region of FMR1 is upregulated in premutation carriers but silenced in full mutation individuals Hum Mol Genet 2007 16 3174 3187 17921506 10.1093/hmg/ddm293

[B82] Khalil AM Faghihi MA Modarresi F Brothers SP Wahlestedt C A novel RNA transcript with antiapoptotic function is silenced in fragile X syndrome PLoS One 2008 3 e1486 18213394 10.1371/journal.pone.0001486 PMC2194623

[B83] Pastori C Peschansky VJ Barbouth D Mehta A Silva JP Wahlestedt C Comprehensive analysis of the transcriptional landscape of the human FMR1 gene reveals two new long noncoding RNAs differentially expressed in Fragile X syndrome and Fragile X-associated tremor/ataxia syndrome Hum Genet 2013 10.1007/s00439-013-1356-6 PMC3898532 24005575

[B84] Edbauer D Neilson JR Foster KA Wang CF Seeburg DP Batterton MN Tada T Dolan BM Sharp PA Sheng M Regulation of synaptic structure and function by FMRP-associated microRNAs miR-125b and miR-132 Neuron 2010 65 373 384 20159450 10.1016/j.neuron.2010.01.005 PMC5018398

